# Alcohol consumption, multiple Lugol‐voiding lesions, and field cancerization

**DOI:** 10.1002/deo2.261

**Published:** 2023-07-03

**Authors:** Chikatoshi Katada, Tetsuji Yokoyama, Tomonori Yano, Haruhisa Suzuki, Yasuaki Furue, Keiko Yamamoto, Hisashi Doyama, Tomoyuki Koike, Masashi Tamaoki, Noboru Kawata, Motohiro Hirao, Yoshiro Kawahara, Takashi Ogata, Atsushi Katagiri, Takenori Yamanouchi, Hirofumi Kiyokawa, Hirofumi Kawakubo, Maki Konno, Akira Yokoyama, Shinya Ohashi, Yuki Kondo, Yo Kishimoto, Koichi Kano, Kanae Mure, Ryuichi Hayashi, Hideki Ishikawa, Akira Yokoyama, Manabu Muto

**Affiliations:** ^1^ Department of Therapeutic Oncology Graduate School of Medicine, Kyoto University Kyoto Japan; ^2^ Department of Health and Promotion National Institute of Public Health Saitama Japan; ^3^ Department of Gastroenterology and Endoscopy National Cancer Center Hospital East Chiba Japan; ^4^ Endoscopy Division National Cancer Center Hospital Tokyo Japan; ^5^ Department of Gastroenterology Kitasato University School of Medicine Kanagawa Japan; ^6^ Division of Endoscopy Hokkaido University Hospital Hokkaido Japan; ^7^ Department of Gastroenterology Ishikawa Prefectural Central Hospital Ishikawa Japan; ^8^ Division of Gastroenterology Tohoku University Graduate School of Medicine Miyagi Japan; ^9^ Division of Endoscopy Shizuoka Cancer Center Shizuoka Japan; ^10^ Department of Surgery National Hospital Organization Osaka National Hospital Osaka Japan; ^11^ Department of Practical Gastrointestinal Endoscopy Faculty of Medicine, Dentistry and Pharmaceutical Sciences, Okayama University Okayama Japan; ^12^ Department of Gastroenterology Kanagawa Cancer Center Kanagawa Japan; ^13^ Department of Medicine, Division of Gastroenterology Showa University Hospital Tokyo Japan; ^14^ Department of Gastroenterology Kumamoto Regional Medical Center Kumamoto Japan; ^15^ Division of Gastroenterology, Department of Internal Medicine St. Marianna University School of Medicine Kanagawa Japan; ^16^ Department of Surgery Kawasaki Municipal Kawasaki Hospital Kanagawa Japan; ^17^ Department of Gastroenterology Tochigi Cancer Center Tochigi Japan; ^18^ Department of Otolaryngology‐Head and Neck Surgery Kyoto University Hospital Kyoto Japan; ^19^ Department of Otorhinolaryngology‐Head and Neck Surgery Kitasato University School of Medicine Kanagawa Japan; ^20^ Department of Public Health Wakayama Medical University School of Medicine Wakayama Japan; ^21^ Department of Head and Neck Surgery National Cancer Center Hospital East Chiba Japan; ^22^ Department of Molecular‐Targeting Prevention Kyoto Prefectural University of Medicine Kyoto Japan; ^23^ Clinical Research Unit National Hospital Organization Kurihama Medical and Addiction Center Kanagawa Japan

**Keywords:** alcohol, esophageal cancer, field cancerization, head and neck cancer, JEC study

## Abstract

The development of multiple squamous cell carcinomas (SCC) in the upper aerodigestive tract, which includes the oral cavity, pharynx, larynx, and esophagus, is explained by field cancerization and is associated with alcohol consumption and cigarette smoking. We reviewed the association between alcohol consumption, multiple Lugol‐voiding lesions, and field cancerization, mainly based on the Japan Esophageal Cohort study. The Japan Esophageal Cohort study is a prospective cohort study that enrolled patients with esophageal SCC after endoscopic resection. Enrolled patients received surveillance by gastrointestinal endoscopy every 6 months and surveillance by an otolaryngologist every 12 months. The Japan Esophageal Cohort study showed that esophageal SCC and head and neck SCC that developed after endoscopic resection for esophageal SCC were associated with genetic polymorphisms related to alcohol metabolism. They were also associated with Lugol‐voiding lesions grade in the background esophageal mucosa, the score of the health risk appraisal model for predicting the risk of esophageal SCC, macrocytosis, and score on alcohol use disorders identification test. The standardized incidence ratio of head and neck SCC in patients with esophageal SCC after endoscopic resection was extremely high compared to the general population. Drinking and smoking cessation is strongly recommended to reduce the risk of metachronous esophageal SCC after treatment of esophageal SCC. Risk factors for field cancerization provide opportunities for early diagnosis and minimally invasive treatment. Lifestyle guidance of alcohol consumption and cigarette smoking for esophageal precancerous conditions, which are endoscopically visualized as multiple Lugol‐voiding lesions, may play a pivotal role in decreasing the incidence and mortality of esophageal SCC.

## INTRODUCTION

The upper aerodigestive tract (UADT) region, which includes the oral cavity, pharynx, larynx, and esophagus, is a highly susceptible area for multiple primary cancers derived from the squamous epithelium. Field cancerization of the UADT region was first proposed by Slaughter et al. to describe multiple squamous neoplastic lesions in this area.^1^ Subsequently, it was found that lifestyle exposure to alcohol consumption, cigarette smoking, betel chewing, poor oral hygiene, and inadequate intake of green and yellow vegetables and fruits, as well as genetic factors for the alcohol metabolism variants aldehyde dehydrogenase 2 (ALDH2, rs671) and ADH1B (rs1229984), synergistically contribute to the risk of multiple squamous cell carcinomas (SCC) in the UADT region.^2^, ^3^, ^4^, ^5^, ^6^

The Japan Esophageal Cohort (JEC) study is a prospective cohort study that enrolled patients with histopathologically diagnosed intramucosal carcinoma after endoscopic resection (ER) for esophageal SCC and no additional treatment. Enrolled patients received surveillance by gastrointestinal endoscopy every 6 months and surveillance by an otolaryngologist every 12 months. Since the enrolled patients were endoscopically resected, the esophagus was preserved, and long‐term survival was expected. The esophageal mucosa could be followed up without the effects of anticancer agents or radiation. At study entry, the health risk appraisal model for predicting the risk of esophageal SCC based on their alcohol flushing (HRA‐F model), alcohol use disorders identification test (AUDIT), mean corpuscular volume (MCV) and grade of Lugol‐voiding lesions (LVL) in the background esophageal mucosa were investigated. All patients were instructed to abstain from drinking alcohol and cigarette smoking through the provision of documentation describing the importance of cessation of drinking alcohol and cigarette smoking. Genetic polymorphisms of ALDH2 and ADH1B were investigated in patients whose consent could be obtained after enrollment.^7^, ^8^

This review focuses on alcohol consumption, multiple LVL, and field cancerization, mainly based on the JEC study, and is a modified version of a previous report in Japanese.^9^

## GENETIC POLYMORPHISMS RELATED TO ALCOHOL METABOLISM

The International Agency for Research on Cancer reported that acetaldehyde associated with alcohol consumption is a Group 1 carcinogen (carcinogenic to humans) with SCC arising in the esophagus or head and neck.^3^ Ethanol is absorbed from the upper gastrointestinal tract and transported to the liver, where it is metabolized by ADH1B to the carcinogenic acetaldehyde. Acetaldehyde is metabolized to noncarcinogenic acetic acid by ALDH2.

About 40% of Japanese have the ALDH2*1/*2 genotype and a few percent have the ALDH2*2/*2 genotype. Individuals with the ALDH2*2/*2 genotype have almost no ALDH2 enzyme activity, and those with the ALDH2*1/*2 genotype have low ALDH2 enzyme activity of about 17%.^10^ Most people with inactive ALDH2 (ALDH2*1/*2 genotype or ALDH2*2/*2 genotype) are weak drinkers who display facial flushing after drinking a single glass of beer but become heavy drinkers if they continue drinking.^11^ When ethanol was administered to ALDH2 knockout mice, acetaldehyde‐induced gene damage occurred, and DNA adducts were produced.^12^ Mutations in the TP53 gene were frequently found in the background esophageal mucosa of esophageal cancer,^7^ and meta‐analyses have shown that both inactive ALDH2 and heavy drinking increased the risk of developing esophageal SCC.^13^

Concerning ADH1B gene polymorphisms, 5%–7% of Japanese are ADH1B*1/*1 positive. The ADH1B*1/*1 genotype has low ADH1B enzyme activity. Individuals with slow‐metabolizing ADH1B (ADH1B*1/*1 genotype) tend to develop alcohol tolerance because of prolonged exposure to ethanol after drinking and is more likely to be heavy drinker. Oral bacteria produce high concentrations of acetaldehyde from ethanol in saliva, and slow‐metabolizing ADH1B individuals are at a higher risk of developing esophageal SCC because ethanol and acetaldehyde remain in saliva for a long time after drinking.^14^ Meta‐analysis also shows that heavy drinkers with slow‐metabolizing ADH1B are at a higher risk of developing esophageal SCC.^15^

Three reports have been published on the association between genetic polymorphisms related to alcohol metabolism and metachronous SCC, which develop after ER for esophageal SCC. Kagemoto et al. reported frequencies of slow‐metabolizing ADH1B and inactive ALDH2 of 26.5% and 69.2%, respectively.^16^ On multivariate analysis, slow‐metabolizing ADH1B (hazard ratio [HR] 2.21; 95% confidence interval [CI] 1.1–4.45) and inactive ALDH2 (HR 3.28; 95% CI 1.28–11.15) were significant predictors of the development of metachronous esophageal SCC or head and neck SCC. Abiko et al. reported frequencies of slow‐metabolizing ADH1B and inactive ALDH2 of 16.5% and 63.9%, respectively.^17^ Multivariate analysis showed that inactive ALDH2 (HR 2.25; 95% CI 1.18–4.59) was a significant predictor of the development of metachronous esophageal SCC or head and neck SCC. Tajiri et al. reported that the frequency of both slow‐metabolizing ADH1B and inactive ALDH2 (double inactivated group) was 19.1% and that metachronous esophageal SCC (*p* < 0.001) and metachronous head and neck SCC (*p* = 0.001) were more common in the double‐inactivated group.^18^ Thus, esophageal SCC and head and neck SCC that develop after ER for esophageal SCC are associated with genetic polymorphisms related to alcohol metabolism.

The JEC study confirmed that slow‐metabolizing ADH1B was a predictor of the development of metachronous esophageal SCC after ER and inactive ALDH2 was also associated with the development of metachronous esophageal SCC.^19^

## LUGOL–VOIDING LESIONS (LVL) IN BACKGROUND ESOPHAGEAL MUCOSA

Muto et al. were the first to report that multiple LVL were related to alcohol consumption and ALDH2 gene polymorphisms.^20^ In patients with esophageal SCC, the population of habitual drinkers with inactive ALDH2 and >100 g of pure ethanol intake per day had a significantly higher risk of developing multiple LVL compared to the population of habitual drinkers with active ALDH2 and <100 g of pure ethanol intake per day (odds ratio [OR] 17.5; 95% CI 1.97–155.59; *p* = 0.01).^21^

**FIGURE 1:**
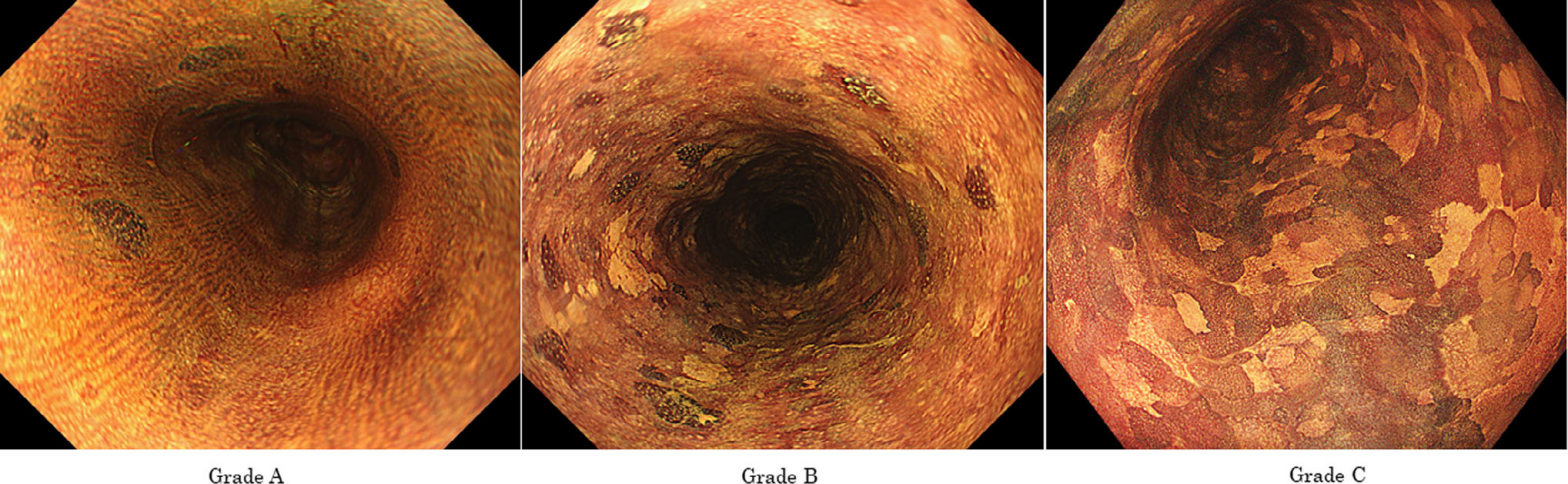
Grading of Lugol‐voiding lesions (LVL). The number of LVL per endoscopic view was counted, and the grading was divided into the following three categories. (a) Grade A: no lesions. (B) Grade B: 1–9 lesions. (c) Grade C: 10 or more lesions.

In the JEC study, the LVL was classified into three grades according to the number of LVL per endoscopic view (grade A, no lesions; grade B, 1–9 lesions; grade C, ≥ 10 lesions) (Figure 1). The 5‐year cumulative incidence of metachronous esophageal SCC at a median follow‐up period of 80.7 months (range 1.3–142.3) was 6.0% for Grade A, 17.8% for Grade B and 47.1% for Grade C, respectively (Grade A vs. B, *p* = 0.022; A vs. C, *p* = 0.0007). The 5‐year cumulative incidence rates of metachronous head and neck SCC were 0.0% for Grade A, 4.3% for Grade B, and 13.3% for Grade C, respectively (Grade A vs. B, *p* = 0.12; A vs. C, *p* < 0.0001).^8^ In the multivariate analysis, Grade C was a significant predictor of the development of metachronous esophageal SCC (HR 8.76; 95% CI 3.02–25.5) and head and neck SCC (HR 3.51; 95% CI 1.34–9.20).^7^

In patients with esophageal SCC, multiple LVL (OR, 61.12; 95% CI, 5.4–691.64, *p* = 0.001), inactive ALDH2 (OR, 16.19; 95% CI, 1.15–228, *p* = 0.04) and current smoker (OR 8.02; 95% CI 1.09–59.22, *p* = 0.04) had significant risk factors for the development of synchronous and metachronous superficial head and neck SCC.^22^ Thus, esophageal SCC and head and neck SCC that develop after ER for esophageal SCC are associated with LVL grade.

## HEALTH RISK APPRAISAL MODEL FOR PREDICTING THE RISK OF ESOPHAGEAL SCC BASED ON THEIR ALCOHOL FLUSHING (HRA‐F MODEL)

Individuals with inactive ALDH2 can be identified by the flushing questionnaire method. The flushing questionnaire method involves questioning a current facial flusher or a former facial flusher immediately after drinking a glass of beer. When current or former flushing individuals were considered to have inactive ALDH2, the sensitivity and specificity of the test were 84.8% and 82.3%, respectively, for the esophageal SCC patients and 90.1% and 88.0%, respectively, for the cancer‐free population. The flushing questionnaire is an established surrogate marker for the ALDH2 genotype.^23^

**FIGURE 2:**
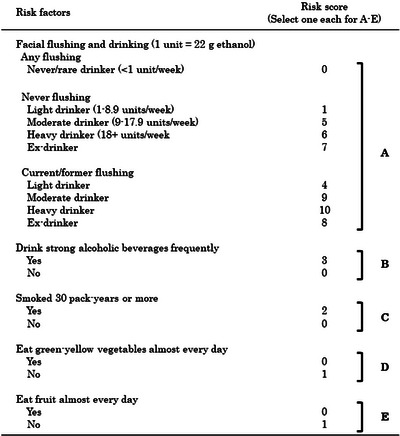
Health risk appraisal model for predicting the risk of esophageal squamous cell carcinoma based on their alcohol flushing (HRA‐F model).

Tobacco is identified as a Group 1 carcinogen by the International Agency for Research on Cancer, and consumption of green and yellow vegetables and fruits is considered a protective factor against the development of esophageal SCC.^3^, ^24^, ^25^ Yokoyama et al. developed an esophageal SCC risk assessment questionnaire (HRA‐F model) that scores alcohol intake history, smoking history, presence of a facial flushing reaction, and vegetable and fruit consumption based on a case‐controlled study^26^, ^27^ (Figure 2). In endoscopic screening, esophageal SCC was detected in 4.27% of the general population with an HRA‐F score of 11 or higher. In the general population aged 50–69 years, esophageal SCC was detected in 2.91% of those with an HRA‐F score of 9 or higher.^28^

In the JEC study, the cumulative incidence of metachronous esophageal SCC was significantly increased in patients with an HRA‐F score of 12 or higher compared to those with an HRA‐F score of less than 12 (HR 2.0; 95% CI 1.21–3.30, *p* = 0.007).^29^ Thus, the risk of developing esophageal SCC is associated with the HRA‐F score.

## MEAN CORPUSCULAR VOLUME (MCV)

MCV tends to increase in heavy drinkers with inactive ALDH2. Macrocytosis of MCV ≥ 106 fl was associated with an increased risk for esophageal SCC (OR, 2.75). Males with both macrocytosis and alcohol flushing had an even higher cancer risk (OR 5.51).^30^

In the JEC study, the cumulative incidence of metachronous esophageal SCC was significantly higher in patients with macrocytosis compared to those without macrocytosis (HR, 2.97; 95% CI, 1.50–5.85, *p* = 0.002). In the multivariate analysis, macrocytosis was a significant predictor of the development of metachronous esophageal SCC (HR 2.23; 95% CI 1.10–4.51).^31^ Thus, the risk of developing esophageal SCC is associated with macrocytosis.

In comparing the grade of LVL in the background esophageal mucosa and MCV, MCV (Mean ± SD) increased progressively from Grade A 93.8 ± 6.3 fl to Grade B 95.8 ± 8.0 fl, to Grade C 97.7 ± 8.7 fl (*P* = 0.047). The frequency of macrocytosis was significantly correlated with 0.0% for Grade A, 6.8% for Grade B, and 11.1% for Grade C (*P* = 0.038).^31^ Thus, MCV reflects the grade of LVL in the background esophageal mucosa.

## ALCOHOL USE DISORDERS IDENTIFICATION TEST (AUDIT)

AUDIT is a screening test developed by the World Health Organization for early preventive intervention of alcoholism. In Japan, a score of 8 or higher is considered hazardous drinking and is indicated for guidance in reducing alcohol consumption, whereas a score of 15 or higher is considered suspicious of alcohol dependence and is recommended for referral to a specialist. In the general male population, 26% of people scored 8 or higher and about 5% scored 15 or higher. In the JEC study, 62.1% scored 8 or higher and 25.4% scored 15 or higher.

In the JEC study, the cumulative incidence of metachronous head and neck SCC was significantly increased in the population with an AUDIT score of 15 or higher compared to those with an AUDIT score of less than 15 (*p* = 0.001). In the multivariate analysis, an AUDIT score of 15 or higher was a significant predictor of the development of metachronous head and neck SCC (HR 6.98; 95% CI 1.31–37.09). However, a high AUDIT score was not a predictor of metachronous esophageal SCC.^32^ Thus, the risk of developing head and neck SCC is associated with an AUDIT score.

In comparing the grade of LVL in the background esophageal mucosa and AUDIT scores, AUDIT scores increased progressively from Grade A to Grade B to Grade C (*p* < 0.0001). The frequency of an AUDIT score of 15 or higher was significantly correlated with 18.0% for Grade A, 20.1% for Grade B and 37.7% for Grade C (*p* < 0.0001).^32^ Thus, an AUDIT score reflects the grade of LVL in the background esophageal mucosa.

## METACHRONOUS HEAD AND NECK CANCER IN PATIENTS WITH ESOPHAGEAL CANCER

Patients with esophageal SCC are more likely to be drinkers and smokers. The International Agency for Research on Cancer has reported the following organs where alcohol consumption causes cancer: the oral cavity, pharynx, esophagus, liver, colon, and female breast.^33^ Cigarette smoking has also been reported to cause cancer of the oral cavity, pharynx, esophagus, stomach, lung, pancreas, colon, liver, kidney, ureter, bladder, cervix, and ovary as well as myeloid leukemia.^34^ Advances in multidisciplinary treatment have led to the long‐term survival of patients with esophageal SCC. Metachronous head and neck SCC after treatment may adversely affect the prognosis and quality of life in patients with esophageal SCC. According to an analysis based on the Japan endoscopy database, head and neck observation was performed during gastrointestinal endoscopy in 57.0% of patients with esophageal SCC.^35^

In the JEC study, the standardized incidence ratio (SIR) of malignancy in patients with esophageal SCC was higher than that in the general population (SIR 3.61, 95% CI 2.83–4.53). The SIR was 25.6 (95% CI 15.6–39.5) for head and neck SCC, 4.65 (95% CI 3.06–6.76) for gastric cancer, 2.13 (95% CI 0.58–5.45) for prostate cancer, 1.86 (95% CI 0.61–4.35) for colon cancer and 1.13 (95% CI 0.31–2.89) for lung cancer.^36^ The 2‐year cumulative incidence of metachronous head and neck SCC was 3.7%, and all patients were cured with preserved laryngeal function. Because surveillance by gastrointestinal endoscopy every 6 months and surveillance by an otolaryngologist every 12 months could detect metachronous head and neck SCC at an early stage, this method of surveillance may become standard follow‐up.^37^

## PREVENTION THROUGH DRINKING AND SMOKING CESSATION

The strong association between inactive ALDH2 and the risk of UADT cancer with drinking alcohol and cigarette smoking has been reviewed in many publications.^38^, ^39^, ^40^, ^41^, ^42^, ^43^ Cigarette smoking also generates elevated concentrations of acetaldehyde and other toxic aldehydes in the saliva. Smoking and drinking together have been shown to lead to a seven‐fold increase in salivary acetaldehyde concentration relative to drinking alone.^44^

In the JEC study, the cumulative incidence of metachronous esophageal SCC was significantly reduced when patients who had habitually drank 22 g or more of pure ethanol per day abstained from alcohol (HR 0.47; 95% CI 0.26–0.85, *p* = 0.013). Reduction in the cumulative incidence of metachronous esophageal SCC was particularly marked in Grade C (HR 0.30; 95% CI 0.13–0.67; *p* = 0.003).^8^ In patients with an HRA‐F score of 12 or higher, abstinence from alcohol also significantly reduced the cumulative incidence of metachronous esophageal SCC (HR 0.37; 95% CI 0.14–0.97, *p* = 0.042).^29^ Some patients who abstained from alcohol or reduced their alcohol consumption improved their LVL grade in the background esophageal mucosa.^45^ For smoking cessation, the short‐term follow‐up did not significantly reduce the cumulative incidence of metachronous esophageal SCC (HR 0.59; 95% CI 0.29–1.18; *p* = 0.13).^7^ However, reanalysis with an extended follow‐up period showed a significant reduction (HR 0.49; 95% CI 0.26–0.91; *p* = 0.024).^8^ Therefore, drinking and smoking cessation is strongly recommended to reduce the risk of metachronous esophageal SCC after treatment of esophageal SCC.

## UNRESOLVED ISSUES AND FUTURE PERSPECTIVES

In the national multicenter survey of 568 superficial head and neck SCC treated by ER in Japan, a total of 131 metachronous cancers arising in other organs were diagnosed in 96 patients (16.9%) in the follow‐up period. Surprisingly, 25 patients (26.0%) died of metachronous cancer arising in other organs. Specific sites of cancer among those 25 patients were esophageal cancer in 8 patients, lung cancer in six patients, colorectal cancer in three patients, gastric cancer in two patients, liver cancer in two patients, bile duct cancer in two patients, duodenal cancer in one patient, and ureteral cancer in one patient.^46^ To improve the outcome of ER for superficial head and neck SCC, it is necessary to detect metachronous cancers arising in other organs at an early stage when they can be effectively treated. We are currently conducting a multicenter prospective cohort study examining the incidence of metachronous cancer in other organs according to the grade of LVL after curative ER of early esophageal cancer (UMIN000018608, JEC‐2 study), as well as a multicenter prospective cohort study in patients who undergo transoral surgery for superficial head and neck cancer (UMIN00000038275, TOS‐J 2 trial). Based on the results of these studies, we expect to be able to establish an effective surveillance method that will allow early detection and improved prognosis of metachronous cancers arising in other organs after ER for esophageal SCC and head and neck SCC.

## CONCLUSIONS

This review focuses on alcohol consumption, multiple LVL, and field cancerization, mainly based on the JEC study. Risk factors for field cancerization provide opportunities for early diagnosis and minimally invasive treatment. Esophageal squamous cell precancerous lesions, which are endoscopically visualized as multiple LVL, can be transformed into cancer by risk factors. Once the risk factors are removed, precancerous lesions have the opportunity to remain still or revert to normal tissue. As the removal of the risk factors is a good choice for treating precancerous lesions, giving up drinking and smoking is beneficial for patients with precancerous conditions. Lifestyle guidance concerning alcohol consumption and cigarette smoking for esophageal precancerous conditions may play a pivotal role in decreasing the incidence and mortality of esophageal SCC.

## CONFLICT OF INTEREST STATEMENT

None.
